# Study of Sr–Ca–Si-based scaffolds for bone regeneration in osteoporotic models

**DOI:** 10.1038/s41368-020-00094-1

**Published:** 2020-09-21

**Authors:** Qianju Wu, Xiao Wang, Fei Jiang, Ziyuan Zhu, Jin Wen, Xinquan Jiang

**Affiliations:** 1 Department of Prosthodontics, Ninth People’s Hospital, College of Stomatology, Shanghai Jiao Tong University, School of Medicine, Shanghai, China; 2 Stomatological Hospital of Xiamen Medical College, Xiamen, China

**Keywords:** Regeneration, Diseases

## Abstract

Bone tissue engineering has emerged as a promising alternative therapy for patients who suffer bone fractures or defects caused by trauma, congenital diseases or tumours. However, the reconstruction of bone defects combined with osteoporosis remains a great challenge for clinicians and researchers. Based on our previous study, Ca–Si-based bioceramics (MSCs) showed enhanced bone formation capabilities under normal conditions, and strontium was demonstrated to be therapeutic in promoting bone quality in osteoporosis patients. Therefore, in the present study, we attempted to enlarge the application range of MSCs with Sr incorporation in an osteoporotic bone regeneration model to evaluate whether Sr could assist in regeneration outcomes. In vitro readout suggested that Sr-incorporated MSC scaffolds could enhance the expression level of osteogenic and angiogenic markers of osteoporotic bone mesenchymal stem cells (OVX BMSCs). Animal experiments showed a larger new bone area; in particular, there was a tendency for blood vessel formation to be enhanced in the Sr-MSC scaffold group, showing its positive osteogenic capacity in bone regeneration. This study systematically illustrated the effective delivery of a low-cost therapeutic Sr agent in an osteoporotic model and provided new insight into the treatment of bone defects in osteoporosis patients.

## Introduction

Currently, osteoporosis has developed into a worldwide chronic disease that more than 200 million people suffer from, and patients often encounter poor bone strength or even bone fracture.^1,2^ The underlying mechanism is mainly attributed to the incongruent biological activities between bone forming and resorption, especially in postmenopausal women who encounter a sharp decrease in serum oestrogen.^3^

Generally, aiming to treat osteoporosis, there are some traditional pharmacological approaches.^4,5^ The representative treatment is strontium ranelate; however, a series of undesirable side effects cannot be ignored for long-term systematic treatment.^6^ Studies have indicated that strontium (Sr) serves as one of the most efficient trace elements for bone metabolism due to its strong bone-seeking properties,^7^ and it can also inhibit osteoclast function.^8,9^ Nonetheless, while studies have mainly focused on the applications of pharmacological agents in the field of osteoporosis, less attention has been paid to site reconstruction of osteoporotic-related bone defects or fractures. Novel therapeutic methods combining bioactive agents within scaffolds for implantation have emerged as promising approaches to address the issues mentioned above.^10,11^ Among biomaterials for bone substitutes, mesoporous silica (MS) materials have attracted significant attention for their intrinsic biocompatibility and decent capability for bone regeneration.^12^ In our previous study, we successfully synthesized Ca–Si-based bioceramics (MSCs) based on the foundation of MS materials, which turned out to be a potential alterative approach for craniomaxillofacial bone regeneration.^13^ However, despite the practicability of MSCs, osteoporosis-related factors could compromise the repair effects of bone substitutes for critical size bone defects, leading to adopted modification for better clinical outcomes. Therefore, the present study tried to incorporate Sr into MSC scaffolds and systematically determine whether Sr-containing MSC scaffolds could be an alternative for the rehabilitation of bone defects in osteoporosis models.

## Results

**Fig. 1:**
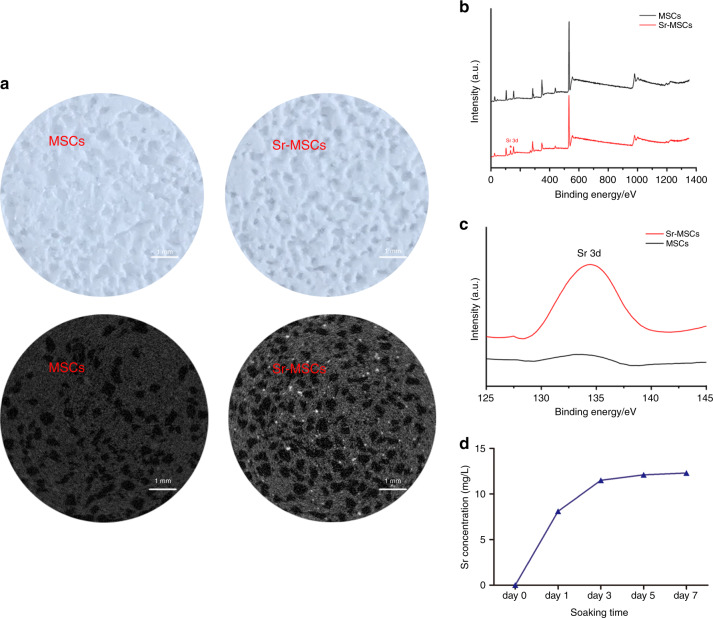
Characterization of the scaffolds. **a** Typical photograph (upper row) and micro-CT images (lower row) of representative MSC or Sr-MSC scaffolds. **b** Surface XPS full spectra of the samples. **c** High-resolution XPS spectra of Sr 3d. **d** The release characteristics of Sr ions

An ordered mesoporous inner structure could be observed for the MSCs by micro-CT. The average mesopore sizes for the MSC and Sr-MSC samples were 402 and 413 μm, and the porosities were estimated to be 63.78 ± 1.8% and 60.38 ± 3.1%, respectively (Fig. 1a and Table 1). According to XPS and further high-resolution analysis, Sr could be detected on the surface of Sr-MSCs (Fig. 1b, c). Subsequently, the release features of Sr ions from the scaffold after immersion in DMEM for 1, 3, 5 and 7 days were investigated. Throughout the whole immersion duration, the release pattern of Sr ions was sustainable from Sr-MSCs and showed an increasing profile (Fig. 1d).

**Table 1:** Porosity and average mesopore size of MSCs and Sr-MSCs scaffolds

Group	Porosity/%	Average mesopore size/μm
MSCs	63.78 ± 1.8	402
Sr-MSCs	60.38 ± 3.1	413

**Fig. 2:**
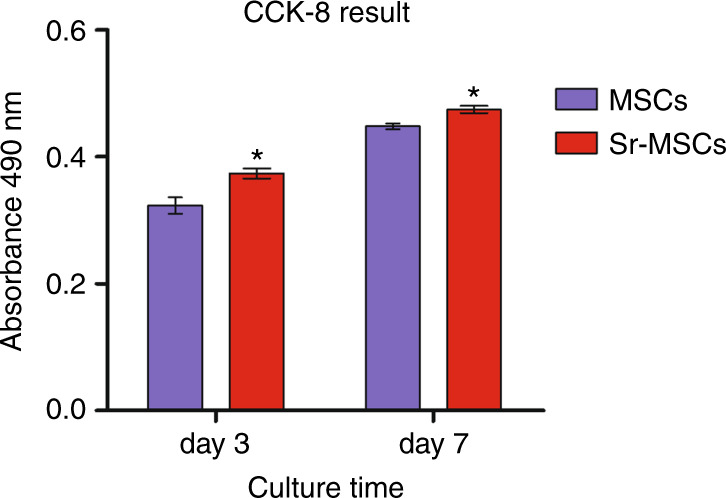
Results of the CCK-8 assay showing the proliferation of OVX BMSCs. Notes: *P < 0.05 versus MSC group

CCK-8 results showed the proliferation viability of Osteoporotic bone mesenchymal stem cells (OVX BMSCs) on various scaffolds (Fig. 2). Notably, an increase in cell proliferation was observed, displaying that the materials did not possess remarkable cytotoxicity and that they were suitable for the following research. Moreover, the proliferation rate of the Sr-MSC scaffolds was significantly higher than that of the MSC group at different time points (*P* < 0.05).

**Fig. 3:**
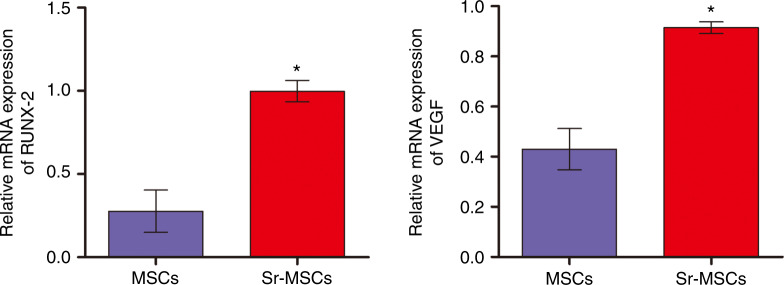
Gene expression of the osteogenesis-related marker RUNX-2 and the angiogenesis-related marker VEGF in OVX BMSCs cultured on various scaffolds. *P < 0.05 versus MSC group

A real-time PCR assay was subsequently employed to evaluate the osteogenic-related and angiogenic markers for OVX BMSCs seeded on each sample. The incorporation of Sr ions into the scaffold remarkably increased the expression of runt-related transcription factor-2 (RUNX-2), implying the potential enhanced osteogenic differentiation of OVX BMSCs. More interestingly, the expression pattern of vascular endothelial growth factor (VEGF) showed dramatic upregulation when compared with MSCs at the same time point (Fig. 3).

**Fig. 4:**
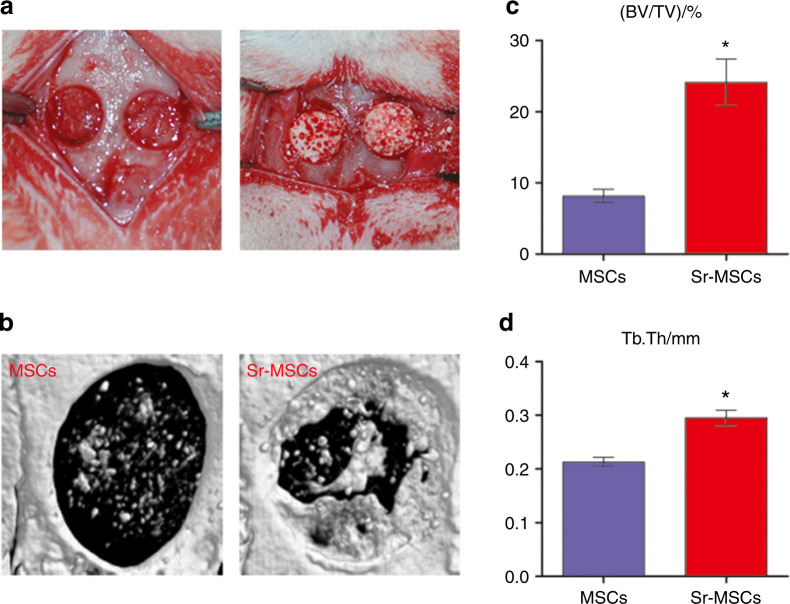
Bone regeneration at 8 weeks. **a** Typical photographs of bone defect models. **b** New bone formation was detected with micro-CT. **c** Quantitative variables for the ratio of bone volume to total volume (BV/TV). **d** The trabecular thickness (Tb. Th) was calculated. Notes: *P < 0.05 versus MSC group

Bone regeneration images were reconstructed at 8 weeks (Fig. 4). These results indicated that a small amount of new bone formation was found in the MSC group. Compared with the MSC group, the Sr-MSC group showed more invasion of new bone into the defect region. The results of the quantitative analysis are displayed. The ratios of bone volume to total volume (BV/TV) were 8.16% ± 1.33% and 24.11% ± 4.65% in the MSC and Sr-MSC groups, respectively, and the latter group was significantly higher than the former group (*P* < 0.05). In addition, the trabecular thickness (Tb. Th) in the Sr-MSC group was also higher than that in the MSC group (*P* < 0.05).

**Fig. 5:**
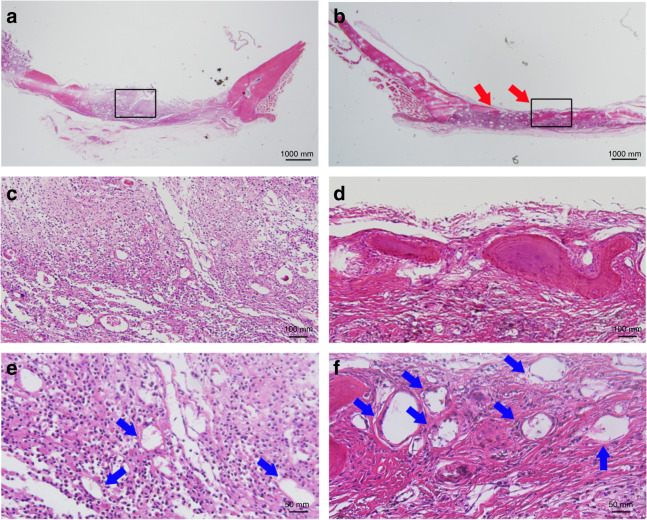
Representative image sections of H&E staining to investigate the repair effects of the defects. The red arrow indicates new bone formation, while the blue arrow represents angiogenesis (**a**, **c**, **e**, MSCs; **b**, **d**, **f**, Sr-MSCs)

The results of histological observation illustrated that in the group of MSCs, only a small amount of bone was found, and the major defect region was filled with fibrous tissue (Fig. 5). Within the defects receiving Sr-MSC scaffolds, more mature bone tissue was observed. Notably, there was a tendency for new blood vessels to emerge in the bone defect area in the Sr-MSC group compared with the MSC group.

## Discussion

Numerous studies have shown that bone tissue engineering grafts have emerged as promising alternatives for bone fracture or defects instead of autologous and allogeneic bone grafts when implanted into a host region.^14–16^ In a previous study, Ca–Si-based bioceramics (MSCs) were successfully fabricated and showed an enhanced bone regeneration effect in a healthy bone defect model. However, reconstruction effects of bone defects caused by osteoporosis remains a severe challenge. The aim of the present study was to take advantage of the therapeutic agent Sr, a main effective element in the well-known anti-osteoporosis drug strontium ranelate, to incorporate Sr into MSCs to evaluate its feasibility in osteoporotic animal models.^9,17^

Sr serves as an essential trace element in the human skeleton. Excessive Sr may lead to cytotoxicity to human organs or bioactivity, making control of the release rate of Sr vital with regard to bone tissue engineering.^18,19^ The current data revealed that sustained release of Sr could be achieved and was much safer than the data that caused adverse cellular effects in the literature.^20^ The technology of loading or incorporating bioactive proteins such as growth factors onto tissue-engineered scaffolds acts as a favourable method to enhance the biological properties of biomaterials. However, high costs, short-term effects and uncertain biological concerns restrict their application in the clinic.^21^ The present study indicated that Sr-modified scaffold could act as a platform for a sustained Sr release pattern to minimize cytotoxicity. Moreover, the CCK-8 assay demonstrated that stem cells seeded on the two scaffolds showed upregulated cell proliferation in a time-dependent manner, and it was remarkable to observe that OVX BMSC proliferation was stimulated by the introduction of Sr. In general, the proliferation of bone-forming stem cells is a vital step that occurs before bone mineralization, and the cellular process is manipulated by the interaction between cells and substrates. Our study showed that the fabricated bone substitutes had no significant cytotoxicity and were endowed with elevated proliferation potential, making the substitutes good foundations for bone regeneration.

A majority of previous studies have reported that Sr in biomaterials could stimulate the differentiation of cells derived from non-osteoporotic models^22–24^; nevertheless, less consideration has been taken into account with respect to the biological interaction between the Sr-incorporated biomaterial tissue engineering approach and the cells harvested from osteoporotic models. In the present study, the incorporation of Sr into MSC scaffolds significantly stimulated the expression level of RUNX-2 in osteoporotic stem cells, implying an underlying therapeutic effect in vivo. Furthermore, the discovery that the expression level of VEGF was simultaneously upregulated was noteworthy. This outcome was in accordance with an investigation that demonstrated that Sr was capable of promoting angiogenic gene expression in human osteoblast-like cells.^25^

Subsequently, critical-sized calvarial defects were prepared in osteoporotic rats to simulate bone defects in osteoporosis, and the potential clinical applicability of Sr-MSC scaffolds was explored in vivo. Additional mineralization was induced, and a higher BV/TV ratio was observed in Sr-MSC scaffolds than in MSC scaffolds. Consistent with the micro-CT findings, the histological assessment showed that a larger area of newly formed bone was regenerated with the Sr-MSC scaffolds. In addition, there was a tendency for blood vessel formation to be enhanced in bony defect areas in the Sr-MSC group compared to MSCs after 8 weeks of implantation. Taking all of these findings into consideration, we speculated that several factors contributed to the enhanced bone regeneration capacity of Sr-MSC scaffolds. Sr ions released from Sr-introduced biomaterials could play an active role in stabilizing the surrounding pH, leading to a better microenvironment and contributing to the facilitation of the osteogenic differentiation of cells.^22^ Moreover, it has been well documented that osteogenesis and angiogenesis are closely linked and regulated by a crosstalk pathway.^26^ Hence, in our study, neovascularization might be accelerated by the expression of VEGF with the help of Sr in bony defects to consequently furnish more oxygen and nutrients essential for bone-forming cells. It was reasonable to speculate that the angiogenesis effect of the nutrient element Sr plays a central role in bone regeneration. Sr may be linked to the stimulation of osteogenic gene expression of stem cells, and the crosstalk between the angiogenesis/osteogenesis pathways may help achieve the current observation.^27^

Our results provide evidence that the Sr-incorporated MSC scaffold was able to promote osteogenic differentiation of OVX BMSCs in vitro and also showed positive osteogenic capacity for bone regeneration in vivo, illustrating the efficient delivery of low-cost therapeutic agents for the treatment of osteoporotic bone defects. However, the in-depth understanding regarding the underlying molecular mechanisms for the biological interaction between Sr-MSC scaffolds and osteoporotic bone-forming cells needs to be further clarified. Additional preclinical studies on a large scale are needed to fully characterize its potential applications in osteoporosis patients.^28^

## Materials and methods

### Fabrication of materials and characterization measurements

The scaffolds were fabricated by a straightforward one-step method as previously described, and whether SrCl_2_ (Sigma, USA) was incorporated or not was denoted as MSCs or Sr-MSCs, respectively.^13^ Micro-CT (SkyScan 1176, Kontich, Belgium) was used to determine the interior structure, and the chemical composition of the samples was investigated by X-ray photoelectron spectroscopy (XPS, Physical Electronics Inc, USA). The different scaffolds were immersed in 10 mL of Dulbecco’s modified Eagle’s medium (DMEM, Gibco, USA) for 1, 3, 5 and 7 days. At each time point, the leaching liquid was acquired for subsequent determination of the released concentration of Sr ions by inductively coupled plasma mass spectrometry (ICP-MS; Nu Instruments, UK).

### Establishment of the osteoporotic animal model

Several Sprague-Dawley rats (female, 3 months old) were selected, and all the following experimental protocols were approved thoroughly by the Animal Care and Experiment Committee, Ninth People’s Hospital. Surgical operations were performed under sterile conditions by employing a minimally invasive approach. As reported in a previous study, bilateral lumbar incisions were made to expose enterocoele by blunt freeing of the subcutaneous connective tissue under general anaesthesia, softly pulling out the ovaries and removing them after ligation of the ovarian vessels. Postoperatively, penicillin was administered to reduce the risk of inflammation.^29^

### OVX BMSC isolation and culture

OVX BMSCs were harvested from the ovariectomized rats 3 months after ovariectomy. Briefly, the osteoporotic rats were sacrificed, and both ends of the femora were cut off to expose the medullary canal. The bone marrow was flushed out with DMEM supplemented with 10% foetal bovine serum (FBS, Gibco, USA), antibiotics (100 U·mL^−1^ streptomycin and 100 U·mL^−1^ penicillin) and 200 U·mL^−1^ heparin (Sigma, USA). Then, the bone marrow mixed with DMEM was centrifuged, and the pellet was suspended and placed in a culture dish. Primary cells were incubated under conventional conditions of 5% CO_2_ and 37 °C. The culture medium was renewed to discard non-adherent cells and the adherent cells, which remained and were cultured, underwent a medium change three times each week. Cells of passages 2–4 were employed for the following studies.^30^

### OVX BMSC proliferation

The proliferation ability of the OVX BMSCs seeded on different scaffolds was determined by Cell Counting Kit-8 (CCK-8) analysis. Briefly, cells (density of 2.0 × 10^4^) were implanted onto the surface of each sample. At 3 and 7 days of culture, CCK-8 solution with a 10% volume of culture medium (DMEM) was added followed by incubation for 1 h at 37 °C to undergo a biological reaction with the cells. The result was interpreted as the absorbance value measured at 490 nm by an ELX ultra microplate reader (BioTek, USA).

### Real-time PCR assay

After 14 days of culture, total cell RNA extraction was carried out by taking advantage of TRIzol (Invitrogen, USA). The harvested RNA was used to synthesize complementary DNA by a PrimeScript 1st Strand cDNA synthesis kit following the manufacturer’s instructions (Takara, Japan). The expression of Runx-2 and VEGF was measured by a reverse transcription polymerase chain reaction (RT-PCR) system (Bio-Rad, USA), and the primer sequences are presented in Table 2. All of the expression values of mRNA were normalized against β-actin, a housekeeping gene, and experiments were performed in triplicate.

**Table 2:** Primers for real-time polymerase chain reaction (PCR)

Gene	Prime sequence (F forward; R reverse)	Product size/bp	Accession number
β-Actin	F: AGGGAGTGATGGTTGGAATG R: GATGATGCCGTGTTCTATCG	107	NM_031004.2
RUNX-2	F: CCGAGACCAACCGAGTCATT R: CACTGCACTGAAGAGGCTGT	114	NM_001278483.1
VEGF	F: TTGAGTTGGGAGGAGGATGT R: TGGCAGGCAAACAGACTTC	115	NM_001110333.1

### Animal surgical procedure

Briefly, a sagittal incision was made on the scalp to expose the calvarium by blunt dissection in the osteoporotic animal model after anaesthesia, and circular defects 5 mm in diameter were fabricated in the calvarial bone. Subsequently, each defect was repaired with MSCs or Sr-MSCs. The incision was sterilized and sutured in layers, and intramuscular injection of antibiotics was administered post-surgically.

### Micro-CT analysis

The animals were sacrificed during week 8, and the skulls from the surgery areas were acquired to analyse the bone volume by micro-CT (SkyScan 1176, Belgium) with a resolution of 18 μm. Reconstruction of the 3-D images to show new bone formation was carried out by 3-D Creator software (Scanco Medical, Switzerland). As previously described, BV/TV and Tb. Th were also analyzed to investigate the quality of new bone formation in each group.

### Histological observations

The skull specimens were acquired and bisected into halves according to the sagittal plane. After dehydration in ascending concentrations of alcohol from 75% to 100%, the specimens were decalcified and embedded into paraffin, and the samples (4 mm thick) were obtained with a microtome (Leica, Germany). Random sections were selected and stained with haematoxylin-eosin (HE) for observation.
